# Evaluation of effect of a vitamin-based barrier cream on the clinical severity of actinic cheilitis: A preliminary study

**DOI:** 10.4317/jced.57013

**Published:** 2020-10-01

**Authors:** Mariana-Sudati Rodrigues, Eduardo-Oliveira Kaefer, Juliana-Tomaz Sganzerla, Humberto-Thomazi Gassen, Rubem-Beraldo dos Santos, Sergio-Augusto-Quevedo Miguens-Jr

**Affiliations:** 1 Lutheran University of Brazil – ULBRA (Graduate Program in Dentistry), Canoas, RS, Brazil; 2 Lutheran University of Brazil – ULBRA (School of Dentistry), Cachoeira do Sul, RS, Brazil

## Abstract

**Background:**

Actinic Cheilitis (AC) is a pathological condition of the labial mucosa considered potentially malignant. The aim of this study was to investigate the effect of treatment of AC with daily use of a vitamin-based barrier cream.

**Material and Methods:**

For this clinical study, 36 participants with lower-lip AC were recruited from three oral medicine services. At baseline, participants were evaluated by clinical examination and clinical severity of AC was classified as grade I to IV. All participants were dispensed a tube of a barrier cream containing vitamins A, D, E and ZnO to apply once nightly for 90 consecutive days. Monthly follow-up was performed to reclassify AC clinial severity. The primary outcome of interest was clinical remission of AC at 90-day follow-up compared to baseline.

**Results:**

Progressive remission of AC lesions was observed as early as the first month and throughout the assessment period (*p* = 0.000). The 3-month period was insufficient for remission of lesions, especially among male participants (*p* = 0.002) and with a longer sun exposure in years (*p* = 0.007).

**Conclusions:**

Daily use of the vitamin-based barrier cream had a promissing positive impact on the severity of actinic cheilitis. However, a 90-day course of treatment was not sufficient to achieve lesions remission. The findings of this study suggest a promising new avenue for the treatment of lower-lip AC.

** Key words:**Actinic cheilitis, vitamins, retinoids, vitamin D, therapeutics.

## Introduction

Actinic cheilitis (AC) is a pathological condition of the labial mucosa, often affecting the lower lip, which is characterized by a chronic inflammatory process caused by labial senility resulting from excessive, continuous ultraviolet radiation (UVR) exposure (1). AC is considered potentially malignant due to the risk of transformation into squamous cell carcinoma (SCC) of the lip (2,3). However, there are no follow-up studies of untreated AC that would allow estimation of annual malignant transformation rates (4). The overall prevalence of QA has been reported to range between 0.45% and 2.4% (2). It is most frequent in fair-skinned males, between the fourth and fifth decades of life, with a history of occupational sun exposure (2,5,6); the prevalence rises to approximately 44% in this population (2,6).

The clinical appearance of AC lesions varies widely, from slight dryness or chapped lips to frank leukoplakia and/or ulcerations. The aspect of these lesions is not always consistent with the histopathological spectrum of epithelial alterations, which include atrophy, hyperkeratosis, and even dysplasia of varying degrees, which portends transformation to SCC (2,3). Another factor to be considered is the delay in diagnosis due to the characteristic asymptomatic nature of AC, which has impacts on both indications for and efficacy of treatment. In addition, AC patients rarely used lip photoprotection as a prevention method (7).

Among several available treatment modalities, the most commonly used options are vermilionectomy (“lip shave”), CO2 laser ablation, photodynamic therapy, and chemotherapeutic agents (8-11). However, patient reports of discomfort and cosmetic and functional sequelae, especially after surgical treatment, have recently prompted the use of minimally invasive therapies. Topical medications are being investigated; these interventions are associated with a lower rate of adverse events (11).

Vitamin-based therapies have been used to reverse cutaneous photodamage and in the treatment of field cancerization (12,13), with particularly promising results for vitamin A analogs. Applied topically, retinoic acid and other metabolites of vitamin A have been used to Fight the signs of photoaging and are capable of reversing the photodamage caused by excessive sun exposure, especially in actinic keratoses; furthermore, they are safe for long-term use (12,14). Topical use of vitamin D (cholecalciferol) and related active ingredients has been shown to reduce cell death and curtail excess proteolytic activity in the epidermis, with consequent improvement in skin hydration and, when combined with other agents, reductions in the number of actinic keratoses and their adverse effects (15-18). Vitamin E (tocopherol), an antioxidant capable of mitigating tissue damage caused by free radicals, appears promising to reduce photoinduced skin damage when used topically (19).

The used of barrier cream containing vitamins and minerals, indicated for the treatment of dermatitis, contains as its active ingredient vitamin D and zinc oxide (ZnO) (19,20). However, there have been no reports on the use of these vitamins on the lip epithelium or in the treatment of AC. Thus, the present study was designed to investigate the clinical effect of treatment of actinic cheilitis (AC) lesions with daily use of a barrier cream containing vitamins A, D, E and ZnO.

## Material and Methods

-Study design and ethical considerations

This clinical preliminary single-arm study was approved by the Research Ethics Committee of the Lutheran University of Brazil (protocol nº 2,751,893) and conducted in accordance with National Health Council Resolution 466/12.

-Study sample and setting

The sample consisted of individuals recruited from three oral medicine services located in different municipalities of south of Brazil.

Sample size calculation, with a statistical power of 80% and a significance level of 5%, was estimated by a mean (SD) difference of 0.63 (0.9) grades of AC severity from baseline after treatment, based on a pilot study by the authors. The minimum sample size was established as 30 participants. To account for possible losses to follow-up, an additional six participants were included (n = 36).

Participants entered the study consecutively, and underwent monthly follow-up until each had individually completed a 90-day treatment period.

-Eligibility criteria

Adult participants (age ≥18 years) of both sexes with a clinical diagnosis of lower-lip AC were deemed eligible. Participants with presence of leukoplakia and/or labial ulcerations, a history or treatment of head and neck cancer, previous history of skin-cancer, diabetes mellitus, immunosuppression, or known allergy to any of the components of the study drug were excluded.

-Intervention and control groups

After an interview to collect sociodemographic (gender, age, education level, household income), behavioral (sun exposure, smoking, alcohol intake), and clinical (medical and dental history) variables, participants underwent clinical examination and photographs were obtained with a 720p HDR digital camera (Apple Inc.) at 240fps for evaluation. Using a scheme adapted from Poitevin *et al.* (21), AC severity was classified as grade I (dryness and/or peeling of the surface of the vermilion of the lips); grade II (atrophy of the vermilion border of the lip with a slightly pale, raised surface and loss of sharpness of the boundary between skin and vermilion border, or presence of a melanotic line); grade III (any of the above, plus presence of rough and scaly areas of hyperkeratosis invading the inner mucosa of the lip); or grade IV (any of the above plus erosion). All procedures were performed by two previously trained examiners. The classification of lesions before the treatment intervention was intended to serve as an individual baseline control for each participant.

For treatment of AC lesions, each participant received a 45g tube of a barrier cream containing vitamins A, D, E and ZnO (Hipoglós®, Johnson & Johnson) and were given verbal and written instructions to apply a thin layer to the upper and lower lips once nightly for 90 consecutive evenings. All were instructed not to eat or drink for at least 1 hour after application. Furthermore, each participant received a stick of Protective Lip Balm (SPF 30) and was instructed to apply it during the day or before any sun exposure.

Throughout the observation period, participants were asked to bring the ointment tube to each study visit for confirmation of treatment adherence. Once treatment had begun, participants returned for follow-up visits at 30, 60, and 90 days. At each visit, two examiners performed all procedures as at baseline. A third examiner an expert in oral medicine, and blinded to clinical information, reviewed all photographs to confirm the classification of AC severity grade in each participant at each time point of assessment.

Photographs were obtained at a standard distance, and resolution and viewed in a dark room on a laptop computer (Apple Inc.). Images from each time point were selected randomly by the third examiner for evaluation every 14 days. Kappa coefficients were calculated to assess intra-examiner agreement. A kappa ≥0.81 (excellent) was considered indicative of a valid result to allow reproducibility of the AC lesion classification method used in the study.

Throughout the observation period, participants whose lesions progressed in severity were immediately excluded from the sample and referred for biopsy.

For analysis of the primary outcome (lesion remission) and estimation of treatment effect, the baseline grade of severity was compared with that observed at the end of follow-up (90 days). The outcome was classified as: complete remission; partial remission; or lesion unchanged (no grade change).

-Data analysis

Data were analyzed by descriptive statistics and generalized estimation equations (GEE) for longitudinal data analysis (observation period), adjusted by Bonferroni multiple comparisons. Analysis of correlation between variables was carried out by Student’s t-test for up to two groups and Spearman’s test, one-way ANOVA, and post-hoc tests for more than two groups. All analyses were performed in IBM SPSS Version 25.0 software. The significance level was set at *p* < 0.05.

## Results

**Figure 1:**
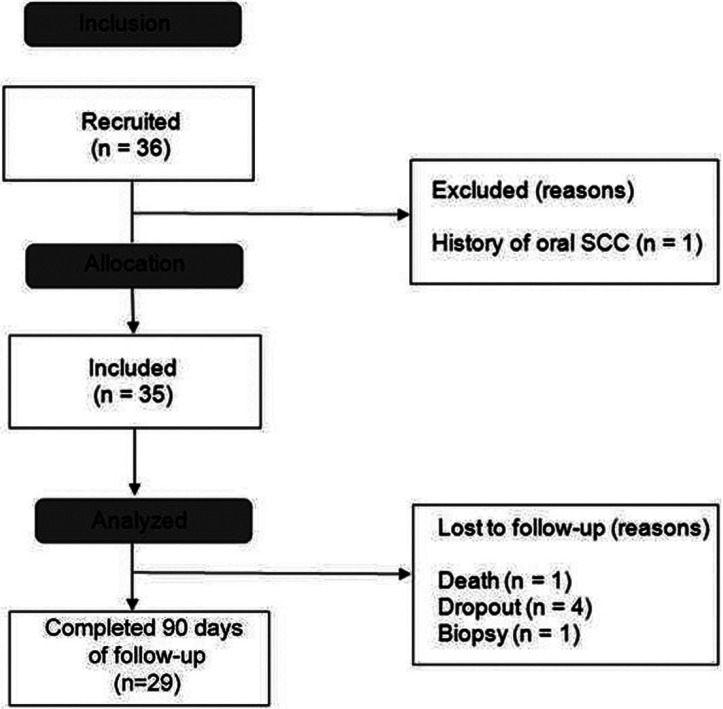
Flowchart of participants selection and follow-up.

Of the 36 participants recruited, 35 met the eligibility criteria and were included in the study. During the observation period, six participants were lost to follow-up: four dropped out, one died, and one participant was excluded at the 60-day visit due to lesion requiring biopsy. Thus, 29 participants completed the study (Fig. 1).

Most participants were male (54.3%), fair-skinned (91.4%), and had worked or otherwise been exposed to work in the sun (65.7%), for at least 8 hours daily (69.56%), over 20 years or longer (52.2%), and had never worn sunscreen (60%). The mean age was 56.66 (± 13.66) years. Many participants had low educational attainment (high school or less; 48.6%), a household income of 1 to 3 times the current minimum wage (37.1%), and most reported some type of comorbidity (e.g., hypertension) (67.5%). Regarding behavioral variables, smoking or former smoking was reported by 91.4% of the sample, while current or past alcohol intake was reported by 20% of participants (Table 1).

**Table 1:**
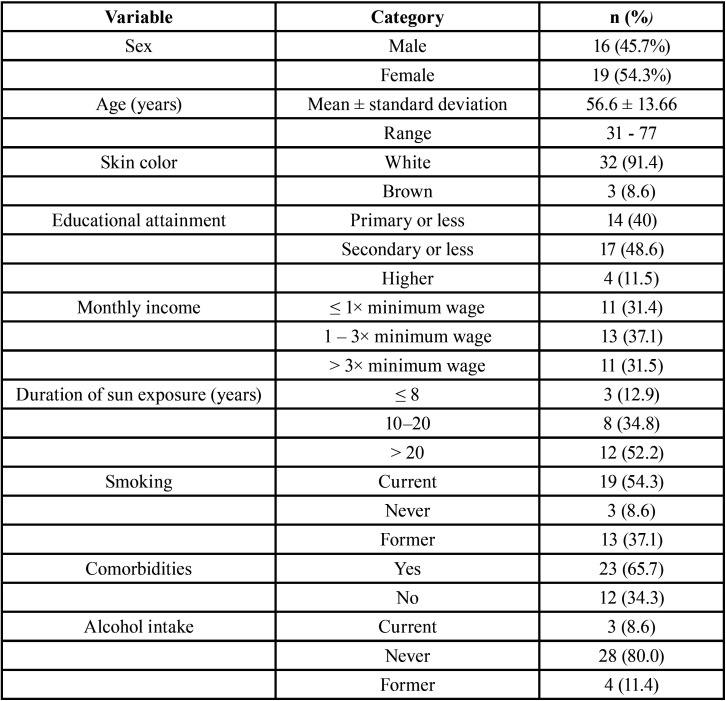
Distribution of sociodemographic, behavioral, and clinical variables of study participants (n = 35).

Intra-examiner agreement on the clinical severity of AC lesions was 94%, while inter-examiner agreement was excellent at κ = 0.915 (95% CI 0.688-1.00). At baseline (n=35), the mean severity of AC lesions was 3.06 (± 0.99), with 45.7% of participants (n = 16) having grade IV and 34,3% (n=12) grades I or II. Over the observation period, mean severity was 2.4 (± 1.10) at 30 days (n = 32), 2.2 (± 1.24) at 60 days (n = 32), and 2.0 (± 1.51) at 90 days (n = 29) (Table 2). A progressive reduction in mean AC severity was thus observed, especially between baseline and 90 days (from 3.06 to 2.03 respectively).

**Table 2:**
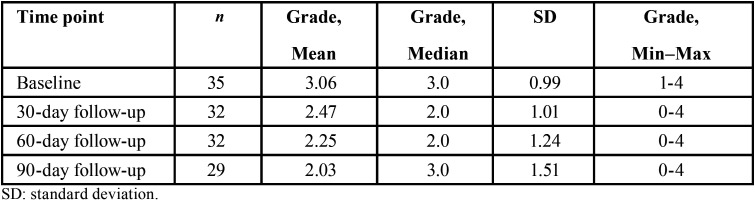
Distribution of severity of actinic cheilitis lesions at baseline and throughout the study period.

Comparison of mean severity at baseline and at 30, 60, and 90 days showed a significant difference in terms of disease regression (*p* = 0.000), starting at 30 days of treatment (Table 3). The largest difference was found on comparison of baseline versus the 90-day end-of-study visit (*p* = 0.000).

**Table 3:**
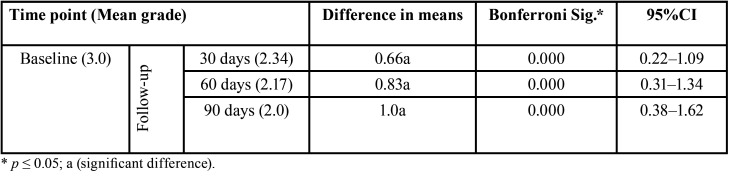
Distribution of mean values and mean differences in actinic cheilitis severity at baseline and follow-up of participants who completed the study period (n = 29).

Of the participants who completed the study (n = 29), 27.6% (n = 8) had experienced no change in AC severity from baseline at 90 days. Three participants (10.3%) had worsening AC and were referred for biopsy. More than half of the sample (62%) had complete (n = 9) or partial (n = 9) remission of AC lesions.

There was a correlation between improvement in AC severity and participant gender, with worse responses being observed in males (*p* = 0.002). Furthermore, the longer the duration of sun exposure in years, the lower the rate of remission (*p* = 0.007). Nevertheless, no correlation was found with participant age (*p* = 0.138) nor with behavioral habits, including smoking (*p* = 0.930).

## Discussion

The results of this study showed that daily use of a barrier cream containing vitamins A, D, E and ZnO had a progressive effect leading to complete or partial remission of lower-lip AC lesions in more than half of the sample (62%), regardless of baseline severity. The 3-month period of observation was not sufficient for complete remission of AC; however, it bears stressing that about 46% of participants had grade IV lesions at baseline. On the other hand, approximately 34% of participants with grade IV lesions at baseline experienced two to four grades of improvement in clinical severity at the end of the 90-day period. In addition, baseline participants with severity grades I–III accounted for the highest proportion of cases achieving complete remission at 90 days.

This effect on the remission of AC lesions may be attribuTable to the components of the barrier cream, especially retinoic acid (vitamin A), which has been shown to have a positive effect on photodamage in UVR-exposed skin (12). Tretinoin, another vitamin A derivative, has been shown to reverse the effects of photoaging, with a 23–60% progressive reduction in actinic keratoses due to cumulative UVR exposure. This effect is attribuTable to a reduction in the stratum corneum and restoration of the dermis within 120 days of treatment (12,13). This may perhaps explain why the 90-day observation period used in the present study was insufficient; use of the barrier cream for a longer period might have allowed the full effect of the retinol component to manifest itself, with the possibility of complete remission of AC lesions.

Vitamin D, another component of the formula, in addition to keeping the lips hydrated and protected, may have acted synergistically with vitamin A. When combined with 5-fluorouracil (5-FU) in the treatment of actinic keratoses, this vitamin has been shown to reduce the number of lesions and minimize adverse events of 5-FU, such as pain, scabbing, and skin ulceration, which also improves treatment tolerability (17).

In the present study, cases classified as grade IV with areas of erosion had a rapid response to treatment. This effect is probably attribuTable to ZnO, another component of the formulation. Zinc compounds are known to increase the potential for keratinocyte migration and collagenolytic activity, in addition to stimulating tissue reepithelization and repair (20). In addition, ZnO-containing creams have been shown to induce complete remission of dermatitis very rapidly (after approximately 4 days of use) (22).

A systematic review (11) compared different treatments for AC lesions and concluded that the combination of imiquimod with photodynamic therapy (PDT) improved clinical appearance in 80 to 100% of cases. However, it is noteworthy that most of the included studies had no formal sample size calculation and enrolled a small number of participants, which may have led to random error and directly impacted the methodological quality and heterogeneity of the available literature regarding the effectiveness of treatments for AC. It should also be noted that, in this review, the use of PDT with imiquimod was implicated in a wide range of adverse effects, including itching, pain, redness, swelling, blistering and crusting, and superficial erosions and ulcerations (8). In the present study, participants only reported superficial lip desquamation in the first days of treatment.

The results of our study cannot be compared to those of invasive treatments for AC. However, there are several less invasive, easy-to-use, low-cost alternatives that can both prevent and treat AC. This is especially relevant considering as verified in the present study—the potential for progressive remission of severe AC lesions, which pose the highest risk of malignant transformation to SCC (21).

In our investigation, we found significant correlations between lower remission rates and longer duration of sun exposure (in years), only among male participants. Possible explanations of this result include the lower adherence of men to treatments that require continuous use (23). Also, one must consider the cumulative effects of UVR exposure to the lip epithelium, which may lead to decreased responsiveness and tissue repair (1,6,24).

Possible limitations of this study include the fact that clinical examination may sometimes underestimate the severity of AC in cases with a less aggressive appearance and varying degrees of undetectable dysplasia. However, this possible confounding factor was minimized by clinical classification of lesion severity, based on well-established criteria (21). Other limitation, only patients of 43 years or older were include in the study.

At the end of the 90-day observation period, despite the loss of follow-up of some participants (n = 6), a number close to that established by the sample size calculation remained. Thus, our study was able to detect a 0.66-grade difference in AC severity between baseline and the end of the study. This can be considered a strength of the present study, as can the prospective follow-up of the participants—which allowed detection of clinical changes at any time and, especially, the possibility of comparing cases before and after the start of the treatment.

Nevertheless, the promising results obtained in this preliminary study with use of a vitamin-based topical therapy for AC, create a perspective for further research. Future studies should focus on longer treatment and follow-up periods and enroll comparator groups to ascertain the efficacy and effectiveness of this treatment.

## Conclusions

Daily topical use of vitamin-based barrier cream has a promising positive impact on the clinical severity of actinic cheilitis. However, a 90-day course of treatment was not sufficient to achieve complete remission. The findings of this study suggest a promising new avenue for the treatment of lower-lip AC.
